# F195. ENRICHMENT OF PATHOGENIC VARIANTS ASSOCIATED WITH TREATABLE GENETIC DISEASES IN LARGE SCHIZOPHRENIA, BIPOLAR AND DEPRESSION COHORTS

**DOI:** 10.1093/schbul/sby017.726

**Published:** 2018-04-01

**Authors:** Venuja Sriretnakumar, Ricardo Harripaul, John Vincent, James Kennedy, Joyce So

**Affiliations:** 1 University of Toronto; 2 Centre for Addiction and Mental Health; 3 University Health Network and Mount Sinai Hospital

## Abstract

**Background:**

Genetic diseases are individually rare but collectively common. Many genetic conditions can mimic mental health disorders, with psychiatric symptoms that are difficult to treat with regular medications. Treatment of the underlying genetic disease can cure the associated psychiatric symptoms or help regular medications work better. Discovery of rare genetic diseases in psychiatric patients would reveal specific treatment options, and give information about the chances of other family members being affected. In this study, we test the hypothesis that psychiatric populations are enriched for pathogenic variants associated with selected treatable inborn errors of metabolism (IEMs).

**Methods:**

Using targeted next-generation sequencing, we screened schizophrenia (n=1132), bipolar (n=719) and major depressive disorder (n=195) patients for variants in genes associated with Niemann-Pick disease type C (NPC), Wilson disease (WD), homocystinuria (HOM) and acute intermittent porphyria (AIP), and compared the frequency of known and predicted pathogenic variants found to 123 136 samples from the gnomAD consortium.

**Results:**

Our study is the first to explore the prevalence of NPC, WD, HOM and AIP gene variants in well-defined psychiatric cohorts. Among 2046 cases (male, n=1106; female, n=940), carrier rates of 0·93%, 0·98% and 0·20% for NPC, WD and HOM were seen, respectively. The carrier rate for NPC was marginally enriched in the SCZ cohort (1·15%) compared to general (95% CI, 0·007 – 0·021; p=0·084) and comparison (95% CI, 1·967 – 5·272; p=5·16e-05) populations. AIP affected rate of 0·29% was observed across the entire psychiatric cohort relative to the general (95% CI, 0·001 – 0·006; p=3·47e-13) and comparison (95% CI, 1·572 – 10·044; p=0·012) populations, an almost 300x enrichment in comparison to what is expected in the general population.

**Discussion:**

An enrichment of known and predicted pathogenic variants associated with NPC and AIP was found in the psychiatric cohort, especially in SCZ patients. The results of this proof-of-principle study support that rare genetic disease variants, such as those associated with treatable IEMs, may contribute to the pathogenesis and treatment responsiveness of psychiatric disorders. Discovering genetic diseases in psychiatric patients will shift how health care is delivered to these vulnerable patients by addressing underlying conditions rather than masking symptoms with medications, and has the potential to especially help patients who don’t respond to regular psychotropic medications. Further studies screening large psychiatric cohorts for pathogenic variants in a large panel of treatable IEM genes will reveal the full impact of such disorders for psychiatric patients.

